# Brief App-Based Cognitive Behavioral Therapy for Anxiety Symptoms in Psychiatric Inpatients: Feasibility Randomized Controlled Trial

**DOI:** 10.2196/38460

**Published:** 2022-11-02

**Authors:** Gaurav Sharma, Lisa Schlosser, Brett D M Jones, Daniel M Blumberger, David Gratzer, M Omair Husain, Benoit H Mulsant, Lance Rappaport, Vicky Stergiopoulos, Muhammad Ishrat Husain

**Affiliations:** 1 Department of Psychiatry University of Toronto Toronto, ON Canada; 2 Centre for Addiction and Mental Health Toronto, ON Canada; 3 Department of Psychology University of Windsor Windsor, ON Canada

**Keywords:** inpatient, mental health, mental disorder, psychiatry, psychiatric, smartphone app, cognitive behavioral therapy, CBT, anxiety, mobile app, mobile health, mHealth, health app, digital health, eHealth, feasibility study, randomized controlled trial, RCT, feasibility, acceptability

## Abstract

**Background:**

Psychiatric inpatients often have limited access to psychotherapeutic education or skills for managing anxiety, a common transdiagnostic concern in severe and acute mental illness. COVID-19–related restrictions further limited access to therapy groups on inpatient psychiatric units. App-based interventions may improve access, but evidence supporting the feasibility of their use, acceptability, and effectiveness in psychiatric inpatient settings is limited. MindShift CBT is a free app based on cognitive behavioral therapy principles with evidence for alleviating anxiety symptoms in the outpatient setting.

**Objective:**

We aimed to recruit 24 participants from an acute general psychiatric inpatient ward to a 1-month randomized control study assessing the feasibility and acceptability of providing patients with severe and acute mental illness access to the MindShift CBT app for help with managing anxiety symptoms.

**Methods:**

Recruitment, data collection, analysis, and interpretation were completed collaboratively by clinician and peer researchers. Inpatients were randomized to two conditions: treatment as usual (TAU) versus TAU plus use of the MindShift CBT app over 6 days. We collected demographic and quantitative data on acceptability and usability of the intervention. Symptoms of depression, anxiety, and psychological distress were measured in pre- and poststudy surveys for preliminary signals of efficacy. We conducted individual semistructured interviews with participants in the MindShift CBT app group at the end of their trial period, which were interpreted using a standardized protocol for thematic analysis.

**Results:**

Over 4 weeks, 33 inpatients were referred to the study, 24 consented to participate, 20 were randomized, and 11 completed the study. Of the 9 randomized participants who did not complete the study, 7 were withdrawn because they were discharged or transferred prior to study completion, with a similar distribution among both conditions. Among the enrolled patients, 65% (13/20) were admitted for a psychotic disorder and no patient was admitted primarily for an anxiety disorder. The average length of stay was 20 days (SD 4.4; range 3-21) and 35% (7/20) of patients were involuntarily admitted to hospital. Small sample sizes limited accurate interpretation of the efficacy data. Themes emerging from qualitative interviews included acceptability and usability of the app, and patient agency associated with voluntary participation in research while admitted to hospital.

**Conclusions:**

Our study benefitted from collaboration between peer and clinician researchers. Due to rapid patient turnover in the acute inpatient setting, additional flexibility in recruitment and enrollment is needed to determine the efficacy of using app-based psychotherapy on an acute psychiatric ward. Despite the limited sample size, our study suggests that similar interventions may be feasible and acceptable for acutely unwell inpatients. Further study is needed to compare the efficacy of psychotherapeutic apps with existing standards of care in this setting.

**Trial Registration:**

ClinicalTrials.gov NCT04841603; https://clinicaltrials.gov/ct2/show/NCT04841603

## Introduction

Inpatient admission plays a crucial role in the treatment of patients with serious mental illness. Patients requiring hospitalization typically present with severe symptoms that require a combination of pharmacological, psychological, and social interventions [1]. While pharmacotherapy is the mainstay of psychiatric inpatient care, most inpatients report that their psychosocial needs are not adequately addressed [2]. For example, prior to the COVID-19 pandemic, only 4% of psychiatric inpatients participated in group psychotherapy and 84% reported being socially disengaged and inactive [3]. This is despite evidence from meta-analyses that inpatient psychotherapeutic interventions are both effective and highly valued by patients [4-6]. COVID-19 pandemic–related social distancing measures have further limited inpatient access to traditional psychosocial interventions.

Anxiety symptoms are frequently targeted in inpatient psychosocial interventions owing to their transdiagnostic ubiquity and relevance [7-9]. Cognitive behavioral therapy (CBT) is an evidence-based intervention for anxiety symptoms and provides a broadly applicable psychotherapy framework suitable for most psychiatric conditions [10,11]. The core principles of CBT can be communicated to people with a range of cognitive abilities, such as children [12] or people with cognitive impairment [13], which is important for inpatients for whom severe symptoms may impede more complex psychotherapeutic modalities. In addition, during inpatient hospitalizations, inpatients have a significant amount of time in a controlled environment with their care team nearby, providing a structure to assist with the acquisition, deployment, and generalization of skills learned in CBT. Group CBT was commonly endorsed in inpatient settings prior to the COVID-19 pandemic to treat a range of symptoms and conditions [3,6]. However, financial and logistical challenges to implementing in-person inpatient group programming have been magnified by the pandemic [14,15]. Innovative solutions to deliver CBT to psychiatric inpatients were being investigated prepandemic and need further investigation urgently [16,17].

Some governments (eg, in Australia) are now providing free access to digital CBT [18]. There is robust and growing evidence for digital CBT for outpatients with mental illness; however, its use on psychiatric inpatient units has not been sufficiently studied [19]. MindShift CBT is a freely available, smartphone-based app developed by Anxiety Canada. There is some preliminary evidence suggesting that this app can be helpful for outpatients with moderate or severe anxiety [20]. While some inpatient programs have started to make personal-use electronics available to the inpatients they serve, studies of specific app-based psychosocial interventions are limited. A study that examined the use of Headspace, a commercially available meditation app, on an inpatient unit had promising feasibility results [17]. However, our review of the literature identified no trials of a CBT app on a psychiatric inpatient unit. Thus, we assessed the feasibility and acceptability of the MindShift CBT app on an inpatient unit and compared its impact to usual inpatient care.

## Methods

### Setting and Participants

This pilot randomized controlled trial was performed at one of the acute general psychiatry units of a large academic psychiatric hospital, the Centre for Addiction and Mental Health (CAMH), which provides secondary and tertiary inpatient care in Toronto, Canada. The study was registered on ClinicalTrials.gov (identifier: NCT04841603). In addition to the clinician researchers, a peer researcher (ie, a researcher with lived experience of mental illness) was involved in recruitment of patients and in the collection and analyses of qualitative data as per best practices for patient involvement in mental health research [21]. This report was prepared according to the CONSORT (Consolidated Standards of Reporting Trials) statement (Multimedia Appendix 1).

All patients admitted to the unit between April 5 and May 5, 2021, were screened by the admitting inpatient team for eligibility and referred to the research team if they assented to hear more about the study. Inclusion criteria were age between 18 and 65 years, fluency in English, a Dynamic Appraisal for Situational Aggression (DASA) score < 3 at the time of referral (which indicates lower acute risk of violence/aggression [22]), and capacity to consent to participation as assessed by the treating team. Exclusion criteria were diagnoses of moderate or severe intellectual disability, learning disability, or neurocognitive disorder, as these participants may have had difficulty navigating the app.

### Ethical Considerations

The study was performed in accordance with the Ethical Principles of Psychologists and Code of Conduct as set out by the British Association for Behavioural and Cognitive Psychotherapies and British Psychological Society, and with the Declaration of Helsinki. All participants provided written informed consent after being provided verbal and written information about the study and before the initiation of any study procedures. The CAMH Research Ethics Review Board approved the protocol, all supplementary documents, and the informed consent form (#116/2020-01). Participants could be withdrawn from the study at their request, the treatment team’s request, or if they experienced significant worsening of symptoms as determined by the research team or treating physicians. Study data were deidentified and anonymized, and participant records were stored in a secure locked drawer in a locked room. Participants were not compensated for their participation in the study. After the trial period, participants in the control group were provided access to the MindShift CBT app.

### Randomization

After provision of informed consent and confirmation of eligibility, participants were randomized without stratification, using the open-source randomizer randomizer.org, to MindShift+treatment as usual (TAU) or TAU. By design, neither the research team nor participants were blinded to group assignment upon commencement of the study.

### Intervention and Control Conditions

After being randomized, participants were involved in the study for 6 days (ie, participants whose length of stay was longer than 6 days were involved for only 6 days). The 6-day study period started on each Tuesday of 4 consecutive weeks. On the first day of the study, each participant met individually with a research team member uninvolved with clinical care on the unit. Participants in both conditions were provided with a tablet to complete the baseline questionnaires (see below). Participants randomized to the intervention (ie, MindShift+TAU) completed an individual introductory session to the app, comprising a review of several sections: the “Home” section, including CBT-based tools such as a fillable thought record and exposure ladder; the “Learn” section containing basic information about anxiety and CBT; the “Quick Relief” section containing meditation exercises; and the “Goals” section, which allows users to set and review progress toward specific and measurable goals. Patients were asked to open the app and use their preferred features for at least 10 minutes and to use the “check in” function daily. This feature requests that users: (1) rate their current mood on a 1-10 Likert scale calibrated with qualitative labels and emoticons; (2) type a response to an open question stating “What’s going on? Describe what’s going on in your life right now and/or what’s on your mind”; and (3) indicate active symptoms of anxiety selected from a checklist. Due to concerns about confidential patient information being shared with others on the unit inadvertently, the tablets used were programmed to require login to the MindShift app each time they were opened. On the third day, each participant was offered a 15-minute session with a clinician or peer member of the research team to address any questions about the study. For participants randomized to the intervention, the use of the app and its core features were also reviewed during these sessions. On the sixth and final day, all participants completed poststudy questionnaires and a debriefing session. Participants in the intervention condition also completed a 30-minute semistructured qualitative interview with a peer or clinician researcher.

All tablets were stored in the nursing station when not in use by patients or when requiring charging. The tablet given to the participants randomized to the control (TAU) condition did not include the MindShift app but had otherwise identical apps installed and accessible, including a video-streaming app and internet browser. TAU on the inpatient unit comprised daily assessments by a psychiatrist, meetings with a social worker as determined by the clinical team, 24/7 access to nursing staff, and pharmacotherapy management. No group psychosocial activities were conducted as part of TAU during the study period due to COVID-19 pandemic–related restrictions. After completing the exit questionnaires on the sixth day of the study, control group participants were offered access to tablets with the MindShift app.

### Measures and Outcomes

The primary outcomes of this trial were indicators of feasibility and acceptability related to the use of the MindShift CBT app in an inpatient setting. Feasibility was assessed with the rates of consent, study completion, and withdrawal, and by completeness of data in pre- and poststudy questionnaires. Acceptability was assessed quantitatively with the Client Satisfaction Questionnaire (CSQ-8), a self-reported scale with 8 items that describes satisfaction with a health service [23]; participants in the intervention condition also completed a user-experience questionnaire for app-based interventions (Multimedia Appendix 2). Given the feasibility-sized sample, changes in symptoms from baseline to the end of the study were classified as secondary quantitative outcomes. The Generalized Anxiety Disorder (GAD-7) scale was used to assess anxiety [24], the 9-item Patient Health Questionnaire (PHQ-9) was used for depressive symptoms [25], and Kessler Psychological Distress Scale (K10) was used as a global assessment of psychological function [26].

### Qualitative Data Analysis

To assess qualitative data, a thematic analysis was applied to transcribed records of the semistructured qualitative interviews using the 15-point checklist of criteria for good thematic analysis published by Braun and Clarke [27]. Transcripts were reviewed and coded independently by the lead clinical researcher and peer researcher, and themes arising from the codes were identified independently. The researchers then met to establish a consensus regarding themes, and items were recorded in alignment with the themes identified by consensus.

### Sample Size, Power, and Statistical Analysis

One of the main goals of this feasibility study was to obtain data that could be used to calculate sample sizes for a future larger, confirmatory trial. Prior research has suggested that a sample size of 12 in each arm suffices for a pilot feasibility study [28]. Given the patient flow on the inpatient unit on which the study was conducted, we planned to conduct the study over 4 weeks with the expectation that this would be long enough to enable randomization of 24 participants.

Statistical analysis was completed using SPSS 28 software. Descriptive statistics, including mean, SD, and range, were calculated for the entire study sample and for each group. The baseline characteristics were compared between the two groups using Kruskal-Wallis tests.

## Results

### Feasibility Measures

**Figure 1:**
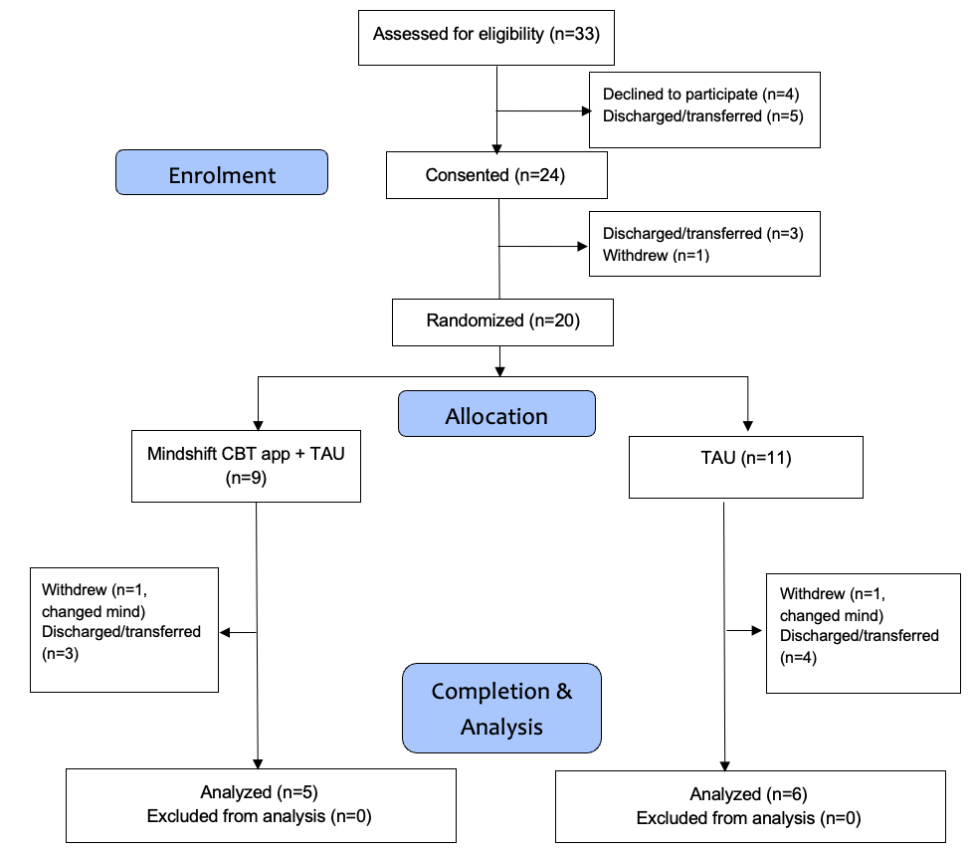
Flow of participant inclusion. CBT: cognitive behavioral therapy; TAU: treatment as usual.

Figure 1 presents the flow of referrals and participation. During the study period (April 5 to May 5, 2021), 33 patients were referred to the study, 5 of whom were discharged or transferred prior to being invited to participate. Of the 28 patients invited to participate, 4 declined, resulting in a consent rate of 24/28 (86%). Of the 24 consented participants, 1 withdrew and 3 were transferred or discharged before being randomized, resulting in a randomization rate of 20/24 (75%). Of the 20 randomized participants, 11 (55%) completed the study and provided pre- and poststudy data that were analyzable. Of the 9 participants who did not complete the study, 2 withdrew (1 in each condition) and 7 were withdrawn because of being discharged or transferred prior to study completion (3 in the MindShift+TAU condition and 4 in the TAU condition).

### Characteristics and Clinical Outcomes of Randomized Participants

Table 1 summarizes the demographic and baseline clinical characteristics of the participants in the two study conditions. Overall, intervention and control condition participants did not show significant differences in baseline characteristics, except for a higher proportion of women in the control condition. Of note, 65% of the participants were admitted with a primary diagnosis of psychotic disorder, and none of the patients who participated was admitted for treatment of a primary anxiety disorder. Overall, 35% of the patients were involuntarily admitted under the Mental Health Act. The mean length of stay of the 20 participants was 20 (SD 4.4, range 3-21) days from the time of admission to the first of either discharge, study completion, withdrawal from the study, or transfer to another unit. Table 1 also presents the clinical outcomes of the 11 participants who completed the study and for whom pre- and poststudy clinical measures were available. The differences in GAD-7, PHQ-9, or K10 scores were not interpretable given the small sample size.

**Table 1:** Demographic and clinical characteristics of the 20 randomized participants.

Characteristic		MindShift+TAU^a^ (n=9)	TAU (n=11)
Age (years), mean (SD)		34.8 (8.0)	29.4 (9.8)
Self-reported sex as female, n (%)		1 (11)	4 (36)
**Self-reported racial/ethnic group, n (%)**			
	White	3 (33)	4 (36)
	Black	2 (22)	2 (18)
	Middle Eastern	1 (11)	1 (9)
	South Asian	1 (11)	1 (9)
	Southeast Asian	1 (11)	1 (9)
	Not reported	1 (11)	2 (18)
Some postsecondary education, n (%)		3 (33)	4 (36)
Annual income below poverty line, n (%)		3 (33)	4 (36)
**Primary diagnosis, n (%)**			
	Schizophrenia-spectrum disorder	6 (67)	6 (55)
	Borderline personality disorder	0 (0)	2 (18)
	Major depressive disorder	0 (0)	2 (18)
	Amphetamine-induced psychotic disorder	1 (11)	0 (0)
	Bipolar disorder	0 (0)	1 (9)
	Opioid use disorder	1 (11)	0 (0)
	Posttraumatic stress disorder	1 (11)	0 (0)
Admitted voluntarily, n (%)		6 (67)	7 (64)
**GAD-7^b^ score, mean (SD)**			
	Prestudy	10.1 (7.3), n=9	8.2 (4.8), n=11
	Poststudy	10.8 (3.3), n=5	9.2 (3.3), n=6
**PHQ-9^c^ score, mean (SD)**			
	Prestudy	11.0 (8.8), n=9	11.1 (5.9), n=11
	Poststudy	13.6 (3.7), n=5	10.8 (3.7), n=6
**K10^d^ score, mean (SD)**			
	Prestudy	27.3 (8.2), n=9	27.3 (8.2), n=11
	Poststudy	27.6 (6.0), n=5	25.8 (6.7), n=6

^a^TAU: treatment as usual.

^b^GAD-7: Generalized Anxiety Disorder Assessment.

^c^PHQ-9: Patient Health Questionnaire.

^d^K10: Kessler Psychological Distress Questionnaire.

### Acceptability Measures

The scores on the CSQ-8 questionnaire (possible range 8-32, higher scores indicate higher satisfaction) completed by the 5 participants in the MindShift+TAU condition (mean 20.2, SD 3.4; range 17-25) indicated overall positive satisfaction with the intervention. Similarly, the scores on the user-experience questionnaire (possible range 0-110, higher scores indicate better usability), available for 4 participants (mean 80.5, SD 27.4; range 46-108), reflected moderate to high user engagement and satisfaction.

### Semistructured Exit Interviews

Four of five participants in the MindShift+TAU condition completed exit interviews, providing information that was grouped into five major themes: feasibility of the intervention in the inpatient setting, usability and patterns of app use, acceptability of the features of the app, a desire to provide feedback and to be seen as active participants during inpatient admissions (which we characterized as agency), and impact of the app on symptoms (see Textbox 1). The benefit of having access to the tablets and app on the inpatient unit was a recurring theme. For instance, one participant commented, “It helped a lot…It gave me access to a tablet and the programs on it, so indirectly I had access to the programs…and do your work as well, so I think it was beneficial.” Users found the app simple to navigate. Frequency of use varied from multiple times per day to just a few times over the week. The “chill zone” feature of the app was the most commented upon and received consistently positive feedback. The participants spontaneously provided suggestions for improvement or additional features, and talked about their participation in research and how their feedback could impact future programming. For instance, one participant commented, “I’ve been asked to participate in the research. This means it’s not just inpatient and that’s it...you could put input in it, you feel that you’re worth something.” There were mixed comments about the impact the app had on symptoms, with some believing use was beneficial, while one participant commented that the app may trigger negative thoughts: “I didn’t like to read too much into it because the info could scare me. It’s like phobias, ‘I’m scared I’m scared!’” Two participants described being confused about the purpose of the pre- and poststudy questionnaires, with one participant conflating the prestudy questionnaires with the CBT app itself.

**Themes and representative quotes emerging from the exit interviews.**

**Theme 1: Feasibility (18 corresponding statements)**

“It helped a lot…It gave me access to, a tablet and the programs on it, so indirectly I had access to the programs…and do your work as well, so I think it was beneficial”

“It was difficult because it took a lot of mental concentration to think through these questions…They’re not like very generic questions like hey, how are you?... so I found myself getting flustered” (in reference to pre- and poststudy surveys)

**Theme 2: Usability (16 corresponding statements)**

“It was very easy to use and click through and enter different…get different information”

“I worked on the app in a different mood and every time I was able to connect”

**Theme 3: Acceptability (21 corresponding statements)**

“It would provide different avenues to have audio abilities, capabilities so you didn’t have to read if you were lethargic, or couldn’t read”

“The chill zone I found was useful, that like mindfulness meditation was great...It’s also very simple. You just sit there and you listen to directions and you chill out”

**Theme 4: Symptoms (11 corresponding statements)**

“I haven’t been able to notice too much of a difference, but I’m sure the app has helped me”

“I didn’t like to read too much into it because the info could scare me. It’s like phobias, ‘I’m scared I’m scared!’”

“The more information I have, the more it helps”

**Theme 5: Agency (22 corresponding statements)**

“It should come back and say you actually did a good job. This is a reward for doing a good job” (in regard to the journal entry capacity of the app)

“I’ve been asked to participate in the research. This mean it’s not just inpatient and that’s it...you could put input in it, you feel that you’re worth something”

“Is me speaking, its it going to the originator who made the program...and will they like to run with it? Or what is the end result from all of this?”

## Discussion

### Principal Findings

This study provides informative data on the feasibility of studying an app-based intervention on an acute psychiatric inpatient unit. A survey of health care provider attitudes toward digital interventions in this setting raised important questions about feasibility, including implementation and suitability for acutely ill patients [29]. Our study supports that the use of digital psychotherapeutic interventions on the inpatient unit requires careful planning and considerations, but could have benefits for an acutely unwell, transdiagnostic population. The high consent rate (24/28, 86%) among inpatients referred to the study demonstrates interest in and willingness to participate in such an intervention. However, the relatively low completion rate (11/20, 55%) emphasizes the challenges of conducting research in acute inpatient settings, where rapid patient turnover can impact the participation and retention of participants. Our goal of obtaining data to calculate sample sizes for a larger confirmatory trial was not achieved because of limitations in our study design and time allotted for data collection, which will inform the design of future trials moving forward. We gathered pre- and poststudy clinical measures of anxiety, depressive symptoms, and psychological distress, but could not accurately interpret these data owing to the small sample size.

While the limited sample size also restricts our ability to draw broad conclusions from the thematic analysis, the quotes provide rich context to reflect patient experiences of the intervention. An unexpected finding was that participants felt empowered by contributing to research and were eager to offer feedback. Voluntary participation in inpatient research projects may stand in psychological contrast to the disempowerment experienced by at least one-third of our participants who were admitted involuntarily. Some participants indicated an intent to download the app on their personal devices after completion of the study and planned to continue to use it after discharge from the hospital. If included in discharge planning, such an intervention could serve as a “transitional object” between inpatient and outpatient settings [30].

A core feature of our study was the collaboration between clinician researchers and a peer researcher for recruitment, data collection, and analysis. However, collaborative efforts must occur earlier in the research process to guide research question development and study design [21]. Compiling research teams of diverse stakeholders, including people with lived experience of mental illness, will be key to designing patient-centered research objectives in the historically coercive inpatient setting.

### Limitations

Of the 20 randomized participants who withdrew from the study, 7 (35%) had to be withdrawn due to being transferred to another more specialized unit or being discharged. Due to limited research personnel, the study operated on a fixed schedule with a 1-week intervention that started on each Tuesday of 4 consecutive weeks, which contributed to low completion rates. A future study would need to use a rolling-entry design in which enrolled participants would be randomized and start the intervention on the day they provide consent. Although the focus of our study was transdiagnostic use of the MindShift app for anxiety symptoms, no participants in the intervention condition had a primary anxiety disorder, which may have impacted the acceptability of the app that is tailored to people with a primary anxiety disorder diagnosis. The requirement to log on to the app at each use presented an additional barrier compared to typical use on personal devices, where users are only required to log in once upon first use of the app and then remain logged in. Additionally, there were unanticipated logistical challenges, which can be generalized to researching other inpatient technology-based interventions, including requiring a system for tablet sign-out, technical troubleshooting, and a need for policies on the inpatient unit about acceptable use of tablets. In addition to the challenges posed to research, these limitations are also relevant to implementation of app-based therapy for clinical use.

### Conclusions

Despite the limitations of this study, our findings support the overall feasibility and acceptability of use of psychotherapeutic apps by inpatients admitted with a variety of psychiatric diagnoses. Specifically, the interactive features of guided meditation and mindfulness in the MindShift CBT app were well-received among our participants. An unintended but important finding was the empowerment described by inpatients from being included in a research study run collaboratively by peer and clinician researchers. Our feasibility trial adds to the limited literature on the use of digital psychosocial interventions on acute psychiatric wards. These interventions could be particularly useful in acute transdiagnostic inpatient settings where tablets or mobile devices are already available, or when traditional group-based psychosocial interventions are not practical (ie, when patients are on infection control precautions). However, it is unclear whether investing in technology-based psychosocial interventions for psychiatric inpatient units would be universally appropriate, or whether these are reasonable substitutes for existing psychosocial treatments. Potential future directions for research include exploring gaps in knowledge about the efficacy of this type of inpatient intervention; exploration of the use of other apps with psychotherapeutic features that are interactive, such as guided meditation and mindfulness; and head-to-head comparison with standard-of-care inpatient group psychotherapy interventions.

## Appendix

Multimedia Appendix 1 CONSORT (Consolidated Standards of Reporting Trials) checklist.

Multimedia Appendix 2 User experience questionnaire.

## Abbreviations

CAMH: Centre for Addiction and Mental Health
CBT: cognitive behavioral therapy
CONSORT: Consolidated Standards of Reporting Trials
CSQ-8: Client Satisfaction Questionnaire
DASA: Dynamic Appraisal for Situational Aggression
GAD-7: Generalized Anxiety Disorder scale
K10: Kessler Psychological Distress Scale
PHQ-9: 9-item Patient Health Questionnaire
TAU: treatment as usual
