# Supplementary material for: Brief App-Based Cognitive Behavioral Therapy for Anxiety Symptoms in Psychiatric Inpatients: Feasibility Randomized Controlled Trial
Source: JMIR Form Res. 2022 Nov 2;6(11):e38460. doi: 10.2196/38460 (PMC9669882; doi:10.2196/38460)
Supplement: Multimedia Appendix 2 [file formative_v6i11e38460_app2.docx]

**Multimedia Appendix 2. User experience questionnaire.**

| **(1) Time to use the App** | | | | | | | | | | | | |
| --- | --- | --- | --- | --- | --- | --- | --- | --- | --- | --- | --- | --- |
| Quick to use | 10 | 9 | 8 | 7 | 6 | 5 | 4 | 3 | 2 | 1 | 0 | Takes too long |
| **(2) Ease of use** | | | | | | | | | | | | |
| Very easy to use | 10 | 9 | 8 | 7 | 6 | 5 | 4 | 3 | 2 | 1 | 0 | Too difficult to use |
| **(3) Visual appeal** | | | | | | | | | | | | |
| Beautiful and engaging | 10 | 9 | 8 | 7 | 6 | 5 | 4 | 3 | 2 | 1 | 0 | Too dull and boring |
| **(4) Consistent (No major change in design, layout or style throughout the App)** | | | | | | | | | | | | |
| Same format throughout | 10 | 9 | 8 | 7 | 6 | 5 | 4 | 3 | 2 | 1 | 0 | No uniformity |
| **(5) Language (Easy to understand with ideal amount)** | | | | | | | | | | | | |
| Easy and short | 10 | 9 | 8 | 7 | 6 | 5 | 4 | 3 | 2 | 1 | 0 | Difficult and too long |
| (**6) Help & support (Can range from online chat to help to written information in the middle to none)** | | | | | | | | | | | | |
| Personalized support | 10 | 9 | 8 | 7 | 6 | 5 | 4 | 3 | 2 | 1 | 0 | None available |
| **(7) Making sense (Does the information make sense)** | | | | | | | | | | | | |
| Easy to understand | 10 | 9 | 8 | 7 | 6 | 5 | 4 | 3 | 2 | 1 | 0 | Makes no sense |
| **(8) Expectation (Is the app able to meet my expectations)** | | | | | | | | | | | | |
| Meets expectations | 10 | 9 | 8 | 7 | 6 | 5 | 4 | 3 | 2 | 1 | 0 | Totally disappointed |
| **(9) Personalized (can the app be personalized)** | | | | | | | | | | | | |
| Personalized to my needs | 10 | 9 | 8 | 7 | 6 | 5 | 4 | 3 | 2 | 1 | 0 | Generic information |
| **(10) Feedback** | | | | | | | | | | | | |
| Feedback available | 10 | 9 | 8 | 7 | 6 | 5 | 4 | 3 | 2 | 1 | 0 | No feedback option |
| **(11) Overall experience of using the app** | | | | | | | | | | | | |
| Excellent user experience | 10 | 9 | 8 | 7 | 6 | 5 | 4 | 3 | 2 | 1 | 0 | Not a good experience |
